# Artificial Intelligence–Assisted Diagnosis of Anterior Cruciate Ligament Tears From Magnetic Resonance Images: Algorithm Development and Validation Study

**DOI:** 10.2196/37508

**Published:** 2022-07-26

**Authors:** Kun-Hui Chen, Chih-Yu Yang, Hsin-Yi Wang, Hsiao-Li Ma, Oscar Kuang-Sheng Lee

**Affiliations:** 1 Institute of Clinical Medicine National Yang Ming Chiao Tung University Taipei Taiwan; 2 Department of Surgery School of Medicine National Yang Ming Chiao Tung University Taipei Taiwan; 3 Department of Orthopedics and Traumatology Taipei Veterans General Hospital Taipei Taiwan; 4 Division of Nephrology Department of Medicine Taipei Veterans General Hospital Taipei Taiwan; 5 Department of Anaesthesiology Taipei Veterans General Hospital Taipei Taiwan; 6 China Medical University Hospital Taichung Taiwan

**Keywords:** artificial intelligence, convolutional neural network, magnetic resonance imaging, MRI, deep learning, anterior cruciate ligament, sports medicine, machine learning, ligament, sport, diagnosis, tear, damage, imaging, development, validation, algorithm

## Abstract

**Background:**

Anterior cruciate ligament (ACL) injuries are common in sports and are critical knee injuries that require prompt diagnosis. Magnetic resonance imaging (MRI) is a strong, noninvasive tool for detecting ACL tears, which requires training to read accurately. Clinicians with different experiences in reading MR images require different information for the diagnosis of ACL tears. Artificial intelligence (AI) image processing could be a promising approach in the diagnosis of ACL tears.

**Objective:**

This study sought to use AI to (1) diagnose ACL tears from complete MR images, (2) identify torn-ACL images from complete MR images with a diagnosis of ACL tears, and (3) differentiate intact-ACL and torn-ACL MR images from the selected MR images.

**Methods:**

The sagittal MR images of torn ACL (n=1205) and intact ACL (n=1018) from 800 cases and the complete knee MR images of 200 cases (100 torn ACL and 100 intact ACL) from patients aged 20-40 years were retrospectively collected. An AI approach using a convolutional neural network was applied to build models for the objective. The MR images of 200 independent cases (100 torn ACL and 100 intact ACL) were used as the test set for the models. The MR images of 40 randomly selected cases from the test set were used to compare the reading accuracy of ACL tears between the trained model and clinicians with different levels of experience.

**Results:**

The first model differentiated between torn-ACL, intact-ACL, and other images from complete MR images with an accuracy of 0.9946, and the sensitivity, specificity, precision, and F1-score were 0.9344, 0.9743, 0.8659, and 0.8980, respectively. The final accuracy for ACL-tear diagnosis was 0.96. The model showed a significantly higher reading accuracy than less experienced clinicians. The second model identified torn-ACL images from complete MR images with a diagnosis of ACL tear with an accuracy of 0.9943, and the sensitivity, specificity, precision, and F1-score were 0.9154, 0.9660, 0.8167, and 0.8632, respectively. The third model differentiated torn- and intact-ACL images with an accuracy of 0.9691, and the sensitivity, specificity, precision, and F1-score were 0.9827, 0.9519, 0.9632, and 0.9728, respectively.

**Conclusions:**

This study demonstrates the feasibility of using an AI approach to provide information to clinicians who need different information from MRI to diagnose ACL tears.

## Introduction

The anterior cruciate ligament (ACL), an important ligament of the knee joint, is a common and devastating sports injury that affects more than 200,000 people in the United States annually [1,2]. The early and proper diagnosis of ACL tears is crucial and can lead to early intervention to prevent subsequent chondral or meniscal damage and early osteoarthritis [3]. A neglected diagnosis can cause longer chronicity of ACL tears at the time of surgery and is positively correlated with the development of osteoarthritis [4]. Arthroscopy can directly visualize the intra-articular lesions of the knee and is the most accurate diagnostic tool for ACL tears [5]. However, this is an invasive procedure with potential surgical risks.

Magnetic resonance imaging (MRI) is a strong, noninvasive tool for detecting ACL tears with high sensitivity and specificity if interpreted by an experienced musculoskeletal radiologist [6,7]. However, reading MR images and making an accurate diagnosis of ACL tears are challenging for less experienced medical personnel.

Graphic identification using deep learning is an important and integral part of artificial intelligence (AI). Using a convolutional neural network (CNN) with repeated input and output data, established algorithms can learn layers of features and repeatedly adjust their neural network and thereby model the complex relationships between medical images and their interpretations [8]. CNNs may be useful in medical imaging tasks; thus, the development of a computer-assisted tool to detect ACL tears from MR images may be helpful in reducing doctor workload, increasing education, reducing misdiagnosis, and enhancing the quality of health care in resource-limited areas [9].

In this study, we aimed to use AI to (1) diagnose ACL tears from complete MR images (for those who were not trained to read knee MRI but nevertheless wanted to diagnose it); (2) identify torn-ACL images from complete MR images that have a diagnosis of an ACL tear (for those who need advanced information after they obtain the result of an ACL tear from the first model); and (3) differentiate torn-ACL and intact-ACL images from the selected MR images (for those who were able to identify the images containing ACL but do not have sufficient confidence in making the diagnosis).

## Methods

### Ethics Approval

This retrospective study was approved by the institutional review board of Taipei Veterans General Hospital (2018-11-005CC).

### Patient Selection and Database

The sagittal MR images of torn ACL (n=1205) and intact ACL (n=1018) from 800 cases and the complete knee MR images of 200 cases (100 torn ACL and 100 intact ACL; torn- and intact-ACL images were extracted, n=335,742) of patients who underwent knee MRI examinations between January 2013 and December 2017 were retrospectively collected for training purposes (training set). The complete MR images of 200 independent cases (100 torn ACL and 100 intact ACL; n=34,914) were used for testing purpose (testing set). The mean age of these patients was 28.1 years and 66.4% (664/1000) were male. The patient population was similar to previous reports on the group with the higher prevalence of ACL tears [10]. We believe these models have routine applications in a majority of patient groups.

Knee MR images excluded patients with the following knee conditions: tumor around the knee, previous knee surgery, multiple ligament injuries, osteoarthritis (Kellgren-Lawrence classification grades 2 to 4), and previous fractures around the knee. MRI examinations were performed on the knee, either in our hospital or in other hospitals, and were then uploaded to our system for a second opinion. There were 6 different MRI scanners used to perform knee examination in our hospital, and we did not restrict the scanner from which we obtained the images. Moreover, we did not identify the scanners in the uploaded images. In this database, for the torn-ACL MRIs, 76.8% (384/500) were performed in our hospital and 23.2% (116/500) were from other hospital; and for the intact-ACL MRIs, 84.6% (423/500) were performed in our hospital and 15.4% (77/500) were from other hospital. Hence, the images used in this study were not restricted to one hospital or a specific MRI scanner.

**Figure 1:**
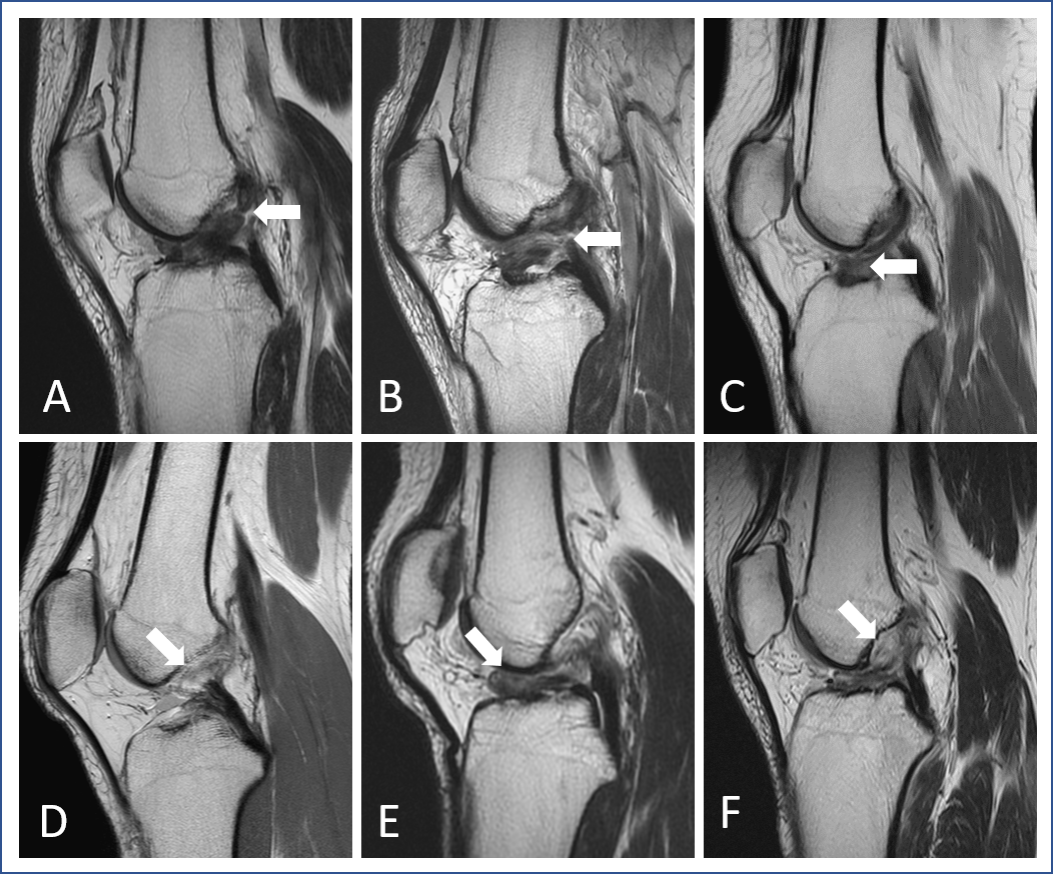
MR images of different torn-ACL patterns. Sagittal proton density images from 6 different patients show variations in the patterns of torn ACL on their respective images: (A) proximal third tear; (B) mid-substance tear; (C) distal third tear; (D) chronic tear with complete ligament resorption, such as ligament disappearance; (E) tear with folded ligament, which may cause extension difficulty; and (F) tear with cyst formation. White arrow: lesion site. ACL: anterior cruciate ligament; MR: magnetic resonance.

**Figure 2:**
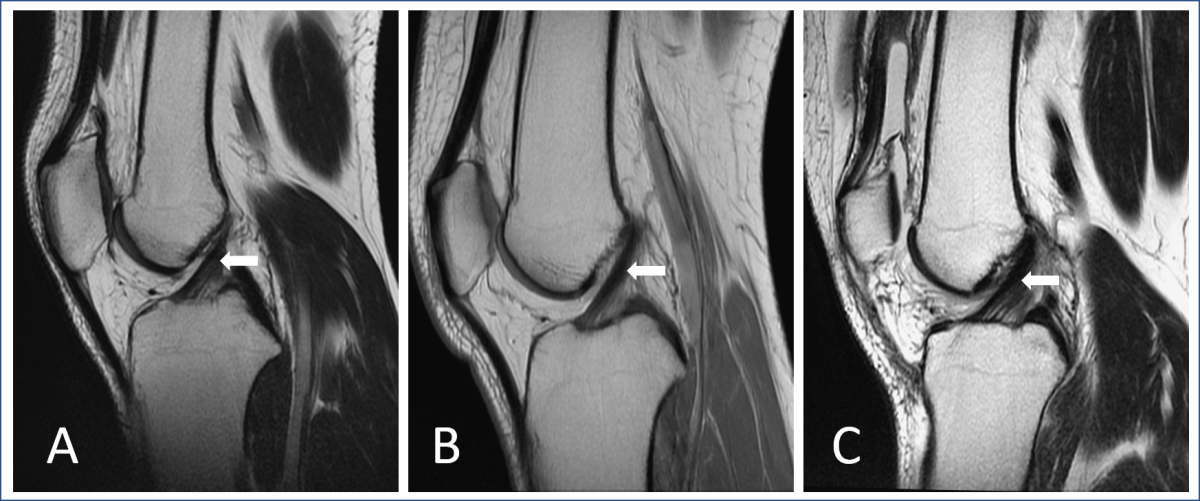
MR images of intact ACL. Sagittal proton density images of 3 different patients are shown. All images show the taut and straight bands parallel to the intercondylar roof with low signal intensity patterns of the intact ACL (white arrow). ACL: anterior cruciate ligament; MR: magnetic resonance.

The determination of a torn-ACL or intact-ACL case was formulated independently by 2 orthopedic doctors and 1 musculoskeletal radiologist who reviewed the MR images and issued the report officially. In addition, torn-ACL cases were also confirmed through arthroscopic examination as all patients with ACL tears underwent arthroscopic ACL reconstruction surgery. All 3 doctors had consistent opinions on the sagittal torn-ACL (Figure 1) and intact-ACL (Figure 2) images.

The first model was for clinicians who were not trained to read knee MR images but wanted to know if the ACL was torn. For this purpose, we first trained a CNN model to differentiate between torn-ACL, intact-ACL, and other images from complete MR images of the knee. The sagittal MR images of torn and intact ACL from 800 cases and the images from 200 complete knee MR images (the torn- and intact-ACL images were extracted), regarded as other images, were used to train and validate the model (Table 1). Cases containing intact-ACL images or both intact- and torn-ACL images were regarded as intact-ACL cases, and cases containing torn-ACL images only were regarded as tear cases. This is similar to the strategy often used by some readers; if an intact-ACL image could be identified among complete MR images, then it might indicate that there is less probability of a torn ACL. Instead, if an intact ACL could not be found when examining the knee MRI of a patient, it would be indicative of a torn ACL.

As the first model did not provide information for identifying torn-ACL images, a second model was developed to identify them from complete MR images that had been diagnosed as ACL-tear case from the first model. Thus, the second model was intended for personnel who needed advanced information on torn-ACL images after obtaining the ACL-tear results. For this purpose, torn-ACL images and other images from 100 ACL-tear cases in the training set were used for training and validation (Table 2).

The third model was used to differentiate between torn-ACL and intact-ACL images from the selected MR images. This model was used by more experienced readers who were able to identify the sagittal images that contained ACLs but needed assistance in making the correct diagnosis. For this purpose, the sagittal MR images of torn and intact ACLs were included for training purposes (Table 3).

**Table 1:** Number of images used for training, validating, and testing the model to differentiate intact-ACL, torn-ACL, and other images from the complete magnetic resonance images.

Classification	Training and validation, n	Test, n
Intact-ACL^a^ images	1018	270
Torn-ACL images	1205	346
Other images	335,742^b^	34,298^c^

^a^ACL: anterior cruciate ligament.

^b^Including sagittal, coronal, and axial images (torn- and intact-ACL images were extracted) from the training set (200 cases).

^c^Including sagittal, coronal, and axial images (torn- and intact-ACL images were extracted) from the test set (200 cases).

**Table 2:** Number of images used for training, validating, and testing the model to identify torn-ACL images from ACL-tear cases.

Classification	Training and validation, n	Test, n
Torn-ACL^a^ images	1205	346
Other images	15,969^b^	16,800^c^

^a^ACL: anterior cruciate ligament.

^b^Including sagittal, coronal, and axial images (torn-ACL images were extracted) from 100 ACL-tear cases in the training set

^c^Including sagittal, coronal, and axial images (torn-ACL images were extracted) from 100 ACL-tear cases in the testing set.

**Table 3:** Number of images used for training, validating, and testing to differentiate between torn- and intact-ACL images.

Classification	Training and validation, n	Test, n
Intact-ACL^a^ images	1018	270
Torn-ACL images	1205	346

^a^ACL: anterior cruciate ligament.

### Image Preprocessing and CNN Model Training by an Automatic Deep-Learning Software

**Figure 3:**
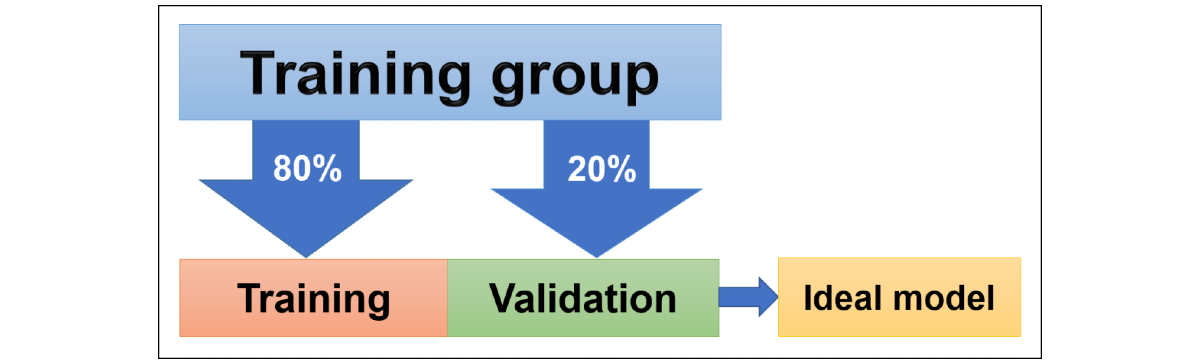
Data organization for model training.

All images were downloaded from the imaging system as a 256 × 256-pixel image in a portable network graphics format and subsequently grouped, as previously mentioned, for training the 3 different CNN models. The AI approach used MAIA automatic deep learning software for medical imaging analyses (version 1.2.0; Muen Biomedical and Optoelectronic Technologies Inc), which was used in a previous study [11]. The CNN model of MAIA was based on EfficientNet-B0, pretrained with ImageNet [12,13]. After inputting the MR images of the training group, 80% of the images were distributed to train and 20% were distributed to validate and find the most ideal CNN model (Figure 3). The MR images were then augmented with horizontal flipping and Gaussian noise [14]. The dropout function and different data augmentation methods were added to prevent the model from overfitting in the data set [15,16]. For hyperparameters in training, the number of epochs was set as 100, the batch size was selected automatically based on memory consumption, and the learning rate was dynamically scheduled through cosine annealing and a 1-cycle policy [17,18]. The network was trained end-to-end using the Adam optimization algorithm, which optimized the cross-entropy as a loss function [19]. For classification, the softmax or sigmoid layer was applied as the output layer in multiclass or binary classification, respectively. The MAIA analysis was performed with Python (version 3.x; Python Software Foundation) and PyTorch (version 1.1.x; Meta AI) on a Windows 10 laptop with GeForce RTX2070 graphic cards (8 GB GDDR6 RAM, GT63 Titan 8SF; MSI).

### CNN Models Performance Evaluation

**Figure 4:**
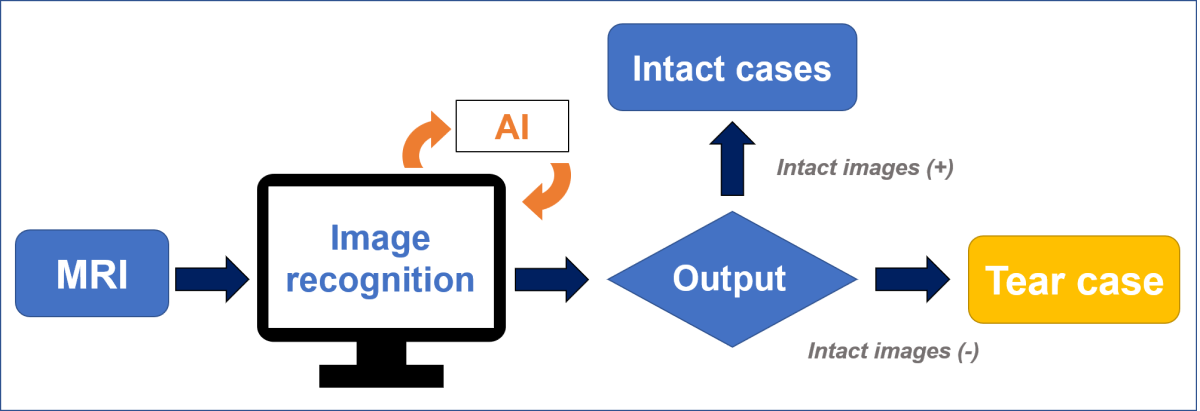
Flowchart of diagnosing ACL tears using the AI approach. ACL: anterior cruciate ligament; AI: artificial intelligence; MRI: magnetic resonance imaging.

**Figure 5:**
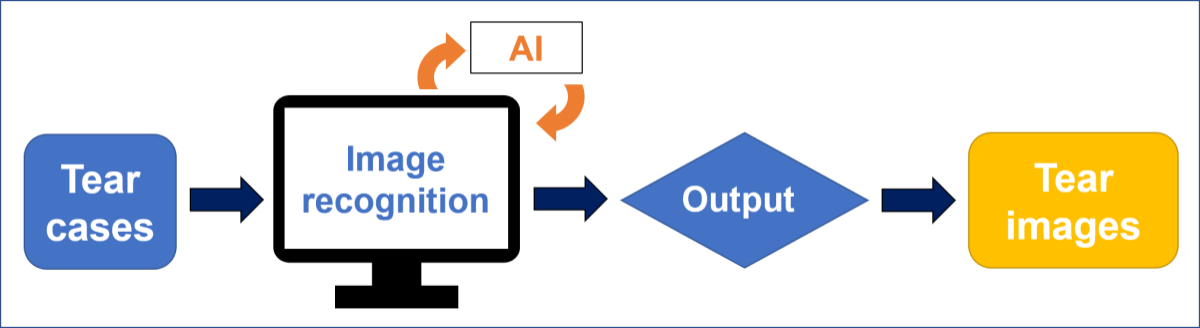
Flowchart of identifying torn-ACL images using the AI approach. ACL: anterior cruciate ligament; AI: artificial intelligence.

**Figure 6:**
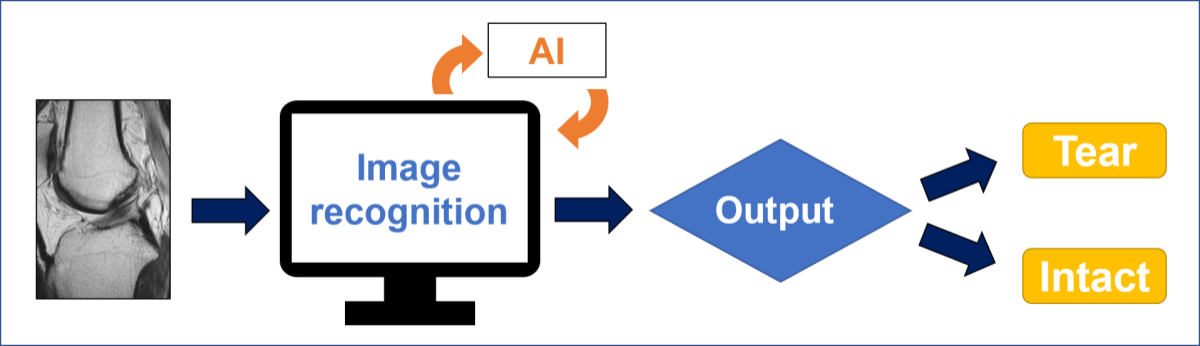
Flowchart of differentiating intact-ACL and torn-ACL images using the AI approach. ACL: anterior cruciate ligament; AI: artificial intelligence.

To evaluate how the model differentiated between torn-ACL, intact-ACL, and other images, the 200 independent cases were used to test the model. To evaluate the accuracy of an ACL-tear diagnosis, cases that were identified as containing intact-ACL images were regarded as intact-ACL cases, and the rest were diagnosed as ACL tears (Figure 4). To evaluate the secondary model of identifying torn images from cases diagnosed with ACL tears, 100 ACL-tear cases from the independent test set were used for testing purposes (Figure 5). To evaluate the third model of differentiating intact-ACL and torn-ACL images from the selected MR images, sagittal MR images labeled as torn and intact ACL from the independent test set were used (Figure 6). Finally, we compared the performance of the first model to diagnose ACL tears with those of orthopedic residents and medical students. For this purpose, 40 randomly selected cases (20 torn and 20 intact) from the test set were used to test differently experienced readers (ie, orthopedic residents and medical students). Complete images were provided to the readers after the removal of personal, clinical, surgical, and institutional information to focus on the reading of the MRI. The residents were split into 3 groups: Group 1 (chief residents and sports fellows), Group 2 (third- and fourth-year residents), and Group 3 (first- and second-year residents). There were 5 participants in each group. We excluded the highest and lowest accuracy results for each group, and the accuracy of each group is the mean accuracy of the 3 readers. The resultant accuracies of the machine and differently experienced readers were compared.

### Statistical Analysis

The effectiveness of the 3 models was evaluated using several metrics, including the accuracy, sensitivity, specificity, F1-score, receiver operating characteristic curve, and the area under the curve, which were calculated using Python. The comparison of the models and doctors with different degrees was performed using SPSS software package (version 22; IBM Corp). Statistical significance was set at *P*<.05, with a 95% CI.

## Results

**Figure 7:**
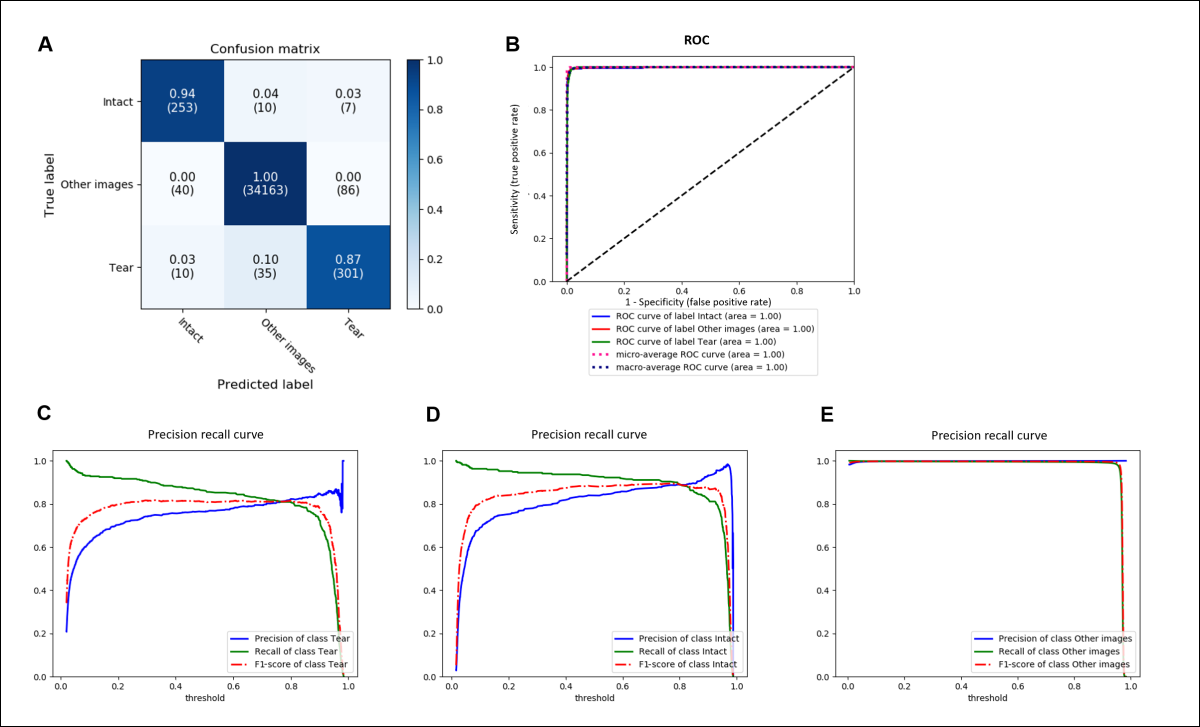
Performance of the model in differentiating torn-ACL, intact-ACL, and other images. (A) Confusion matrix; (B) ROC curve of the model; (C) Precision recall curve for identifying torn-ACL images; (D) Precision recall curve for identifying intact-ACL images; and (E) Precision recall curve for identifying other images (images without torn or intact ACL). ACL: anterior cruciate ligament; ROC: receiver operating characteristic.

**Figure 8:**
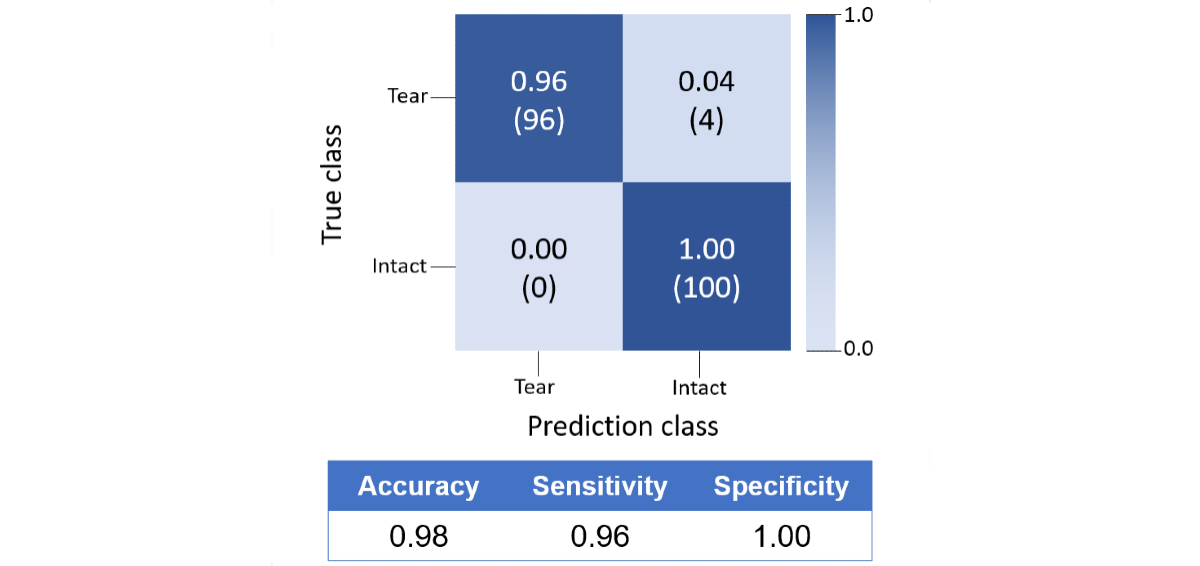
Classification matrix for diagnosing ACL-tear cases. ACL: anterior cruciate ligament.

**Figure 9:**
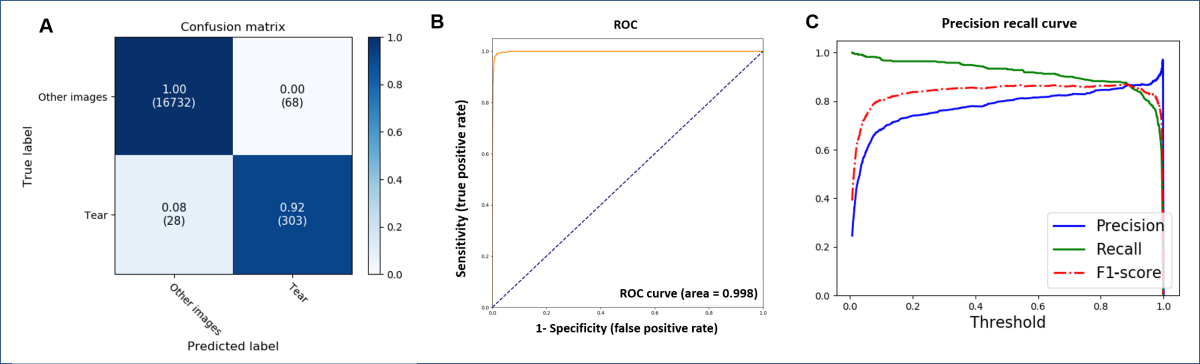
Performance of the model in identifying torn-ACL images from complete MRI images with an ACL-tear diagnosis. (A) Confusion matrix; (B) ROC curve of the model; and (C) Precision recall curve. ACL: anterior cruciate ligament; ROC: receiver operating characteristic.

**Figure 10:**
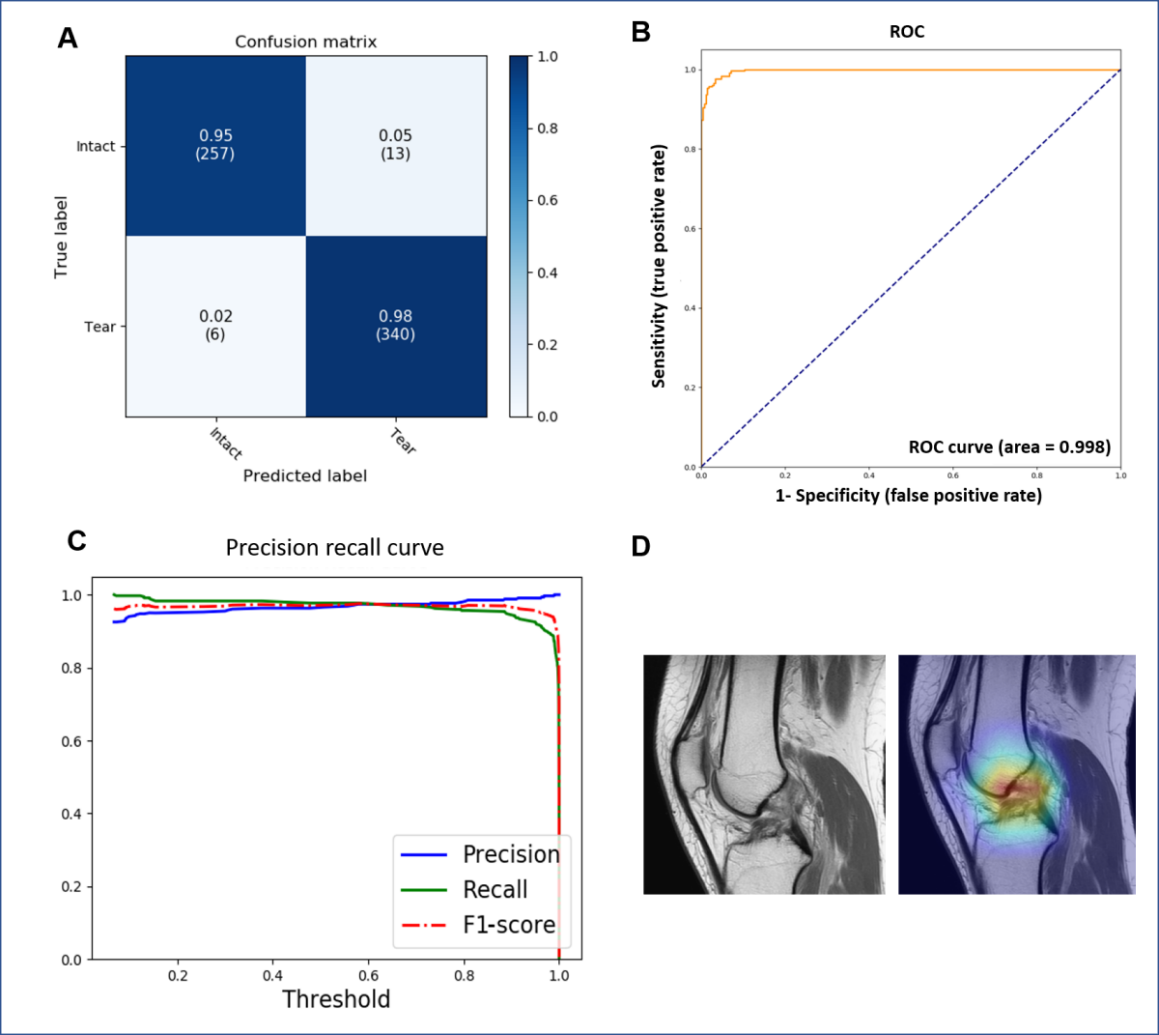
Performance of the model in differentiating between intact-ACL and torn-ACL images. (A) Confusion matrix; (B) ROC curve of the model; (C) Precision recall curve; and (D) torn-ACL image identified (left) and its representative heat map (right). ACL: anterior cruciate ligament; ROC: receiver operating characteristic.

The accuracy of the model that differentiated between torn-ACL, intact-ACL, and other images was 0.9946. The sensitivity, specificity, precision, and F1-scores were 0.9344, 0.9743, 0.8659, and 0.9980, respectively (Table 4 and Figure 7). The accuracy of ACL diagnosis was 0.96 (Figure 8). The accuracy of the model identifying torn-ACL images from the complete images of ACL-tear cases was 0.9943. The sensitivity, specificity, precision, and F1-scores were 0.9154, 0.9660, 0.8167, and 0.8632, respectively. (Table 4 and Figure 9). The accuracy of the model that differentiated torn- and intact-ACL images was 0.9691. The sensitivity, specificity, precision, and F1-scores were 0.9827, 0.9519, 0.9632, and 0.9782, respectively (Table 4 and Figure 10).

The accuracy of the first model and the differently experienced orthopedic residents and medical students for the diagnosis of ACL tears is shown in Table 5. When using the 40 randomly selected cases from the test set for reading comparison, the results showed a significantly higher reading accuracy for the model than those of the less experienced residents and medical students.

**Table 4:** Validation and test results for the 3 models.

Model	Torn-ACL^a^, intact-ACL, and other images differentiation		ACL-tear image identification			ACL-tear or intact images differentiation	
	Validation	Test	Validation	Test	Validation		Test
Accuracy	0.9947	0.9946	0.9959	0.9943	1.0000		0.9691
Sensitivity	0.9702	0.9344	0.9834	0.9154	1.0000		0.9827
Specificity	0.9884	0.9743	0.9969	0.9660	1.0000		0.9519
Precision	0.9647	0.8659	0.9595	0.8167	1.0000		0.9632
F1-score	0.9674	0.8980	0.9713	0.8632	1.0000		0.9728

^a^ACL: anterior cruciate ligament.

**Table 5:** Accuracy of the model and the differently experienced orthopedic residents and medical students in the diagnosis of anterior cruciate ligament tears in 40 randomly selected magnetic resonance imaging cases.

Reader	Accuracy, mean	*P* value^a^
Machine	0.975	Reference^b^
Group 1: chief residents and sports fellows (n=3)	0.888	.13
Group 2: third- and fourth-year residents (n=3)	0.817	.02
Group 3: first- and second-year residents (n=3)	0.742	.003
Medical students (n=3)	0.708	.001

^a^*P* values were based on statistical analyses using the chi-squared test. Statistical significance was set at *P*<.05.

^b^The accuracy of machine reading was used as a reference.

## Discussion 

### Principal Findings

This study demonstrates the feasibility of using an AI approach to diagnose ACL tears from complete MR images with 96% accuracy, identify torn-ACL images from ACL tear cases with 99.4% accuracy, and differentiate intact-ACL and torn-ACL images from the selected MR images with 96.9% accuracy. The model also demonstrated a significantly higher diagnostic accuracy than orthopedic residents in training and medical students.

MRI is a highly accurate tool for evaluating ACL tears, with an accuracy, sensitivity, and specificity of more than 90% [20,21]. In a complete MR scan, the knee should ideally be imaged in 3 orthogonal planes: sagittal, coronal, and axial slices. During the examination, the patient was positioned supine in the scanner, with the knee relaxed in mild flexion and slight external rotation (5°-10°). This position enables the ACL to be orthogonal to the sagittal plane of imaging [22]. Therefore, of all 3 planes, sagittal plane images show the ACL most clearly, especially with T2-weighted sequences [23]. When reading knee MR images in clinical practice, sagittal images are more commonly used to evaluate the condition of the ACL than the other planes. For this reason, we chose to use the sagittal images of the intact or torn ACL as the target for the AI approach to develop the 3 models.

In a normal knee, the ACL is between the lateral femoral condyle and the anterior midportion of the tibia and attaches the anterior to the tibial spine. Sagittal MR images appear as a taut and straight band parallel to the intercondylar roof (Blumensaat line) and have low signal intensity on T1- and T2-weighted images (Figure 2). However, compared to intact-ACL images, there are many variations in the torn-ACL sign on the MR images. These variations include discontinuity in the different parts of the ligament (proximal, midsubstance, or distal) [24], abnormally increased signal intensity, and abnormal morphology, such as a wave, fold, or angulation. In chronic tears, the ACL can even be nonvisualized owing to the resorption of the torn ligament (Figure 1). Thus, the variable appearance of torn-ACL images makes them more complicated to read than intact-ACL images. In the first model, the results showed that the model had less accuracy in identifying torn-ACL images than intact-ACL images (0.87 vs 0.94). There was more misprediction of other images as torn-ACL images, and many of these mispredictions occurred in the intact-ACL cases, identifying both intact-ACL and torn-ACL images as intact-ACL cases (19 cases). However, there was less misprediction of other images as intact-ACL images in ACL-tear cases (4 cases). All the results reflected the variations in torn-ACL images. Accordingly, for the purpose of diagnosing ACL-tears, cases containing intact-ACL images were regarded as intact cases because the model identified them with a higher accuracy. The other cases without intact-ACL images were regarded as tear cases. By using this principle to exclude ACL-tear cases, the accuracy of the diagnosis of ACL tears could reach 96%, which is comparable to many studies using different AI approaches [25-27]. This method can be helpful for personnel who are not trained to read the knee MRI but want to know if the ACL is torn. In addition to diagnosing ACL tears, this study also demonstrates the feasibility of identifying ACL images from complete MR images of ACL-tear cases and differentiating intact- and torn-ACL images with a good accuracy and F1-score. These models can be useful for various user needs.

A total of 40 cases were randomly selected from the test set for the reading of the model and from differently experienced residents and medical students. The images provided for each case were complete MRI examinations, which included all planes and sequences. The results showed that the accuracy of the model in diagnosing ACL-tear cases was significantly higher than that of medical students and orthopedic residents in training. Reading MR images to identify ACL tears is relatively routine for attending orthopedic surgeons or radiologists. However, for less experienced readers, the model may provide a useful reference when they are uncertain of the diagnosis.

In this study, we did not extract images from only 1 specific MR scanner. This is because, in daily practice, a hospital may have multiple scanners, and sometimes a physician may need to read MR images from an unknown scanner from another hospital. The MR images for this study were obtained using 6 different MR knee scanners in our institute, which were obtained from 2 different companies and purchased in different years. In addition to MR images that were obtained in our hospital, images were also taken from other hospitals and uploaded to our image system when the patients came for a second opinion or asked for surgery. Therefore, our data set comprised images from different scanners, and it was less likely that the model would learn some artifacts from the scanners that are not related to the ACL condition. We demonstrated that the models can perform well for an independent test set that contains MR images from different scanners.

### Comparison With Prior Work

Using a deep-learning approach to detect ACL tears has been reported with an accuracy exceeding 95% in many studies using different AI approaches [25-28]. Nonetheless, there were some novelties in this study that we consider to be comparable for their use in daily practice. First, we extracted images from heterogeneous MR scanners. In previous studies, only 1 or 2 scanners were used; however, it is uncommon that there are only 1 or 2 MRI scanners in an institution. Thus, developing a deep-learning algorithm that is trained with images from different MR scanners may better represent real-world situations in many hospitals. For the independent test set, we used the complete images of the MRI examination, and there was no restriction on the protocol used by the scanner, which is different from previous studies. Second, we used a different approach to diagnose the ACL injuries. We excluded the cases containing the intact-ACL images, which were identified by the AI approach, to diagnose ACL-tear cases with an accuracy of 96%. Third, we developed 3 different models for users with different purposes: (1) to diagnose ACL tears from complete MR images; (2) to identify torn-ACL images from complete MR images with a diagnosis of ACL tears; and (3) to differentiate intact-ACL and torn-ACL MR images from the selected images. Users with different experiences require different types of help. These 3 models are tailored to assist users with different needs by providing them with relevant information using an AI approach, which has not been previously reported.

### Limitations

Our study has several limitations. First, we did not label the partially torn–ACL images. Partial tears of the ACL are more difficult to diagnose than complete tears, and the accuracy of these diagnoses is poor on MR images [29]. Thus, we did not use the images of partial tears for training or testing in this study. However, should a partial tear case be input into the model, the model could diagnose the case as an ACL tear because this model cannot identify an intact-ACL image. This finding may alert the user that the case is a torn-ACL case, and the case may need to be double-checked by an orthopedic specialist. Second, we used only sagittal torn-ACL and intact-ACL images for the diagnosis of ACL tears. Considering that the images of other planes can also assist in the diagnosis, adding the other planes of images into the training might increase the reading accuracy. Third, we did not record the details of the MR scanners, because the information of the scanners of the images taken from other hospitals could not be identified.

### Conclusions

This study demonstrates the feasibility of using an AI approach to diagnose ACL tears from a complete MR image (with 96.0% accuracy), identify torn-ACL images from ACL-tear cases, and differentiate intact-ACL and torn-ACL images from the selected MR images. These models may serve as clinical decision support systems for diagnosing ACL injuries for clinicians with different experiences and purposes in reading knee MRIs.

## Abbreviations

AI: artificial intelligence
ACL: anterior cruciate ligament
CNN: convolutional neural network
MOST: Ministry of Science and Technology
MRI: magnetic resonance imaging
